# Intrinsic bias in breast cancer gene expression data sets

**DOI:** 10.1186/1471-2407-9-214

**Published:** 2009-06-29

**Authors:** Jonathan D Mosley, Ruth A Keri

**Affiliations:** 1 Department of Pharmacology, Case Western Reserve University School of Medicine, Cleveland, USA; 2 Division of General Medical Sciences–Oncology, Case Western Reserve University School of Medicine, Cleveland, USA

## Abstract

**Background:**

While global breast cancer gene expression data sets have considerable commonality in terms of their data content, the populations that they represent and the data collection methods utilized can be quite disparate. We sought to assess the extent and consequence of these systematic differences with respect to identifying clinically significant prognostic groups.

**Methods:**

We ascertained how effectively unsupervised clustering employing randomly generated sets of genes could segregate tumors into prognostic groups using four well-characterized breast cancer data sets.

**Results:**

Using a common set of 5,000 randomly generated lists (70 genes/list), the percentages of clusters with significant differences in metastasis latencies (HR p-value < 0.01) was 62%, 15%, 21% and 0% in the NKI2 (Netherlands Cancer Institute), Wang, TRANSBIG and KJX64/KJ125 data sets, respectively. Among ER positive tumors, the percentages were 38%, 11%, 4% and 0%, respectively. Few random lists were predictive among ER negative tumors in any data set. Clustering was associated with ER status and, after globally adjusting for the effects of ER-α gene expression, the percentages were 25%, 33%, 1% and 0%, respectively. The impact of adjusting for ER status depended on the extent of confounding between ER-α gene expression and markers of proliferation.

**Conclusion:**

It is highly probable to identify a statistically significant association between a given gene list and prognosis in the NKI2 dataset due to its large sample size and the interrelationship between ER-α expression and markers of proliferation. In most respects, the TRANSBIG data set generated similar outcomes as the NKI2 data set, although its smaller sample size led to fewer statistically significant results.

## Background

Over the past decade, a large number of global gene expression data sets of human breast cancers have become publicly available [1-6]. These data sets have provided a wealth of information for the generation and testing of biological and clinical hypotheses [7]. Clinical and pathological factors with relevance to breast cancer are extensively characterized, and the prognostic significance of these factors is reflected in these publicly available data sets. These factors include tumor grade, Her2 and estrogen receptor (ER) expression [8]. Whether gene expression data contributes additional prognostic information beyond what is offered by these clinical factors is debated [9,10]. Gene expression profiles associated with individual clinical hallmarks have also been described. For instance, a large set of genes is associated with ER gene expression [5,11]. In addition, the molecular basis of grade has also been examined with results showing a strong relationship between histological grade and tumor proliferation [6,12]. Indeed, the consistent prognostic efficacy of a proliferation signature is now well established [13,14].

The strong inter-relationships between clinical features, gene expression patterns and prognosis has led to the postulate that, depending upon the underlying relationship between the clinical and prognostic factors in a given data set, prognostic gene expression signatures may simply function as a proxy measure for these established clinical variables. For example, Gruvberger et. al. showed that a gene expression signature derived from the van't Veer data set [15], for which ER status was a strong predictor of the incidence of metastasis, was not predictive of metastasis in a data set for which this relationship did not exist [16]. This observation led the authors to propose that derivation of future prognostic gene signatures stratify analyses by ER status in order to adjust for the known association between gene expression and ER status. This suggestion has been variably implemented, but is often ignored. Another consequence of the association between prognosis and large sets of correlated genes is that a large number of predictive gene lists can be derived by selecting different members of predictive clusters of correlated genes. This phenomenon can occur even when gene selection adheres to a standardized protocol due to variations such as the specific tumors used in training sets (subsets of tumors used to derive prognostic lists) [10,17].

While breast cancer gene expression data sets have considerable commonality in terms of their data content, the populations that they represent and the data collection and analysis methods can be quite disparate. The advantage of this heterogeneity is that it provides an opportunity to test the robustness of a hypothesis across multiple populations represented in these data sets [18]. However, a potential disadvantage of this heterogeneity is that systematic differences between data sets, unrelated to analytical approaches, may create sources of bias that impact their intrinsic likelihood of confirming a given hypothesis.

To gain insight into the extent of potential systematic differences in obtaining statistically significant results across breast cancer gene expression data sets, we ascertained whether there were intrinsic differences in the likelihood of observing an association between the expression of a selected set of genes and metastasis using four well-characterized data sets. To minimize bias in our approach, an unsupervised clustering algorithm was used to segregate tumors into one of two clusters based on the expression levels of randomly selected sets of genes (30–400 genes/set). These clusters were then compared for differences in metastasis latencies. We found that one data set, the Netherlands Cancer Institute (NKI2) set of young women with early stage disease, was considerably more likely to give a significant finding when examining either all tumors or only ER positive tumors, as compared to the other data sets examined. Factors that contributed to an increased likelihood of a random gene list being predictive of metastasis within a given data set were 1) the number of tumors analyzed and 2) the inter-relationships between ER expression, proliferation and metastases. We suggest that these intrinsic differences between the data sets should be considered in the design and analysis of future studies incorporating gene expression data.

## Methods

### Previously published microarray data sets

Global gene expression and clinical data (including estrogen receptor status and metastasis recurrence latencies) were analyzed in four independent, publicly available breast cancer gene expression data sets. The Netherlands Clinical Institute (NKI2) data set contains data on 295 women with early stage breast cancer (downloaded from http://www.rii.com/publications/default.htm) [4,19]. The Wang data set contains gene expression data on 296 women with lymph node negative disease [1] (GEO series GSE2034). The KJX64 and KJ125 data sets contain data on 189 women, 64 of which were treated with tamoxifen, with primary operable invasive breast cancer (GEO series GSE2990) [6]. The TRANSBIG data set contains data for 183 untreated women from the Bordet Institute (GEO series GSE7390) [20,21].

All probes from each data set were used in the analyses except in the NKI2 data set where only the probes where there was complete data on more than 291 of the 295 subjects were used (n = 24,023 probes). Missing values for genes in the NKI2 set were imputed using the "impute" option in the FastClus procedure. Gene expression data for the NKI2 data set were given as log10 expression ratios, while data from the TRANSBIG and KJX64/KJ125 sets were log2 expression values. Expression values were log2 transformed in the Wang gene expression data set. Each gene probe in each data set was mapped to a unigene cluster ID using the SOURCE database (source.stanford.edu).

For each gene expression data set, a new data set containing a single set of expression data for each unique unigene cluster ID was created. In the Wang, Miller and KJX64/KJ125 data sets, the expression values for each probe was set to have a median value of 0 and standard deviation of 1. In instances where there were multiple gene probes with a common unigene cluster ID, the median expression value of all the common probes was used. There were 11,318 genes with unique unigene identifiers that were common to all four data sets.

### Clustering using random gene lists

Random lists of genes of various sizes (30 to 400 genes per list) were generated by simple random sampling (Surveyselect procedure). For each randomly-generated gene list, tumors were separated into two groups using an unsupervised hierarchical clustering procedure that was based on a correlation matrix derived from standardized centroid components for cluster assignment (Varclus procedure). The two groups represent the first bifurcation of the clustering hierarchy. Random gene lists were also generated using subsets of genes that were identified as being associated with overall metastases latencies. The subsets of genes utilized in this analysis were those that had a relatively modest proportional hazards p-value of less than 0.1 in a Cox regression analysis.

### Survival analyses

All survival analyses were based on 5-year metastatic recurrence latencies. All subjects not experiencing metastasis within 5 years were censored at that time point. Cox proportional hazards regression models were used to ascertain differences in latencies between groups assigned by hierarchical clustering (PHReg procedure). Univariate proportional hazard ratios and p-values are reported for all analyses and represent the differences in risk between the relatively "poor" prognosis group as compared to a relatively "good" prognosis group. In univariate analyses examining the association between ER status and latencies, hazard ratios represent the change in risk for ER positive tumors, as compared to ER negative tumors. Multivariable models adjusted for ER-α expression used the ESR1 probe in the NKI2 data set and the "205225_at" gene probe in other data sets.

### Determining cluster assignment association with ER status

For each pair of clusters created by the hierarchical clustering procedure, the percentage of all ER negative and ER positive tumors contained in each cluster was computed. The maximum value of the ratio of the ER negative percentage to the ER positive percentage among the two clusters was then computed. If ER positive and negative tumors were assigned to clusters in equal proportions, this ratio would be approximately 1. Otherwise, this ratio would be greater than 1, indicating that the clusters contained a relatively disproportionate number of ER negative tumors, as compared to ER positive tumors.

### Global adjustment of gene expression data

To eliminate correlations between all genes in the NKI2 data set and either ER-α or proliferation genes, the data were globally adjusted by fitting a least-squares regression line to each probe in the dataset and then computing the residuals (GLM procedure) [14]. Each probe was the dependent variable and either ER-α or a proliferation gene was the continuous independent variable. The residuals (adjusted values) for each probe represent the new expression values for that probe. This new adjusted value is no longer linearly correlated with the independent variable. The gene selected to adjust each data set for proliferation was based on a previously published analysis and represents the gene probe that was the most predictive member of a set of correlated proliferation-associated genes linked with an increased risk of developing metastases [14]. The specific genes used in each data set were UBE2C (NKI2 and TRANSBIG), HMMR (KJX64/KJ125) and RACGAP1 (Wang).

### Determining the total variance explained by a gene cluster

The total variance for a data set was computed by summing up the variances for each gene in the data set. To determine the variance explained by globally adjusting a data set for a gene, the total variance of the adjusted data set was computed and the percent change of this value relative to the unadjusted value was determined. The proportion of the proliferation-associated variance explained by adjusting for ER-α was calculated using the equation:

where Var(EP) is the percent change in variance after sequentially adjusting a data set for ER-α and proliferation and Var(E) and Var(P) are the percent changes in variance after individually adjusting for either the ER-α or proliferation genes, respectively.

### Statistical packages

All calculations were performed using SAS version 9.1 (SAS Institute, Cary, NC). All statistical tests were two-sided, and a p-value less than 0.01 was considered statistically significant.

## Results

### Intrinsic bias in gene expression data sets

**Figure 1:**
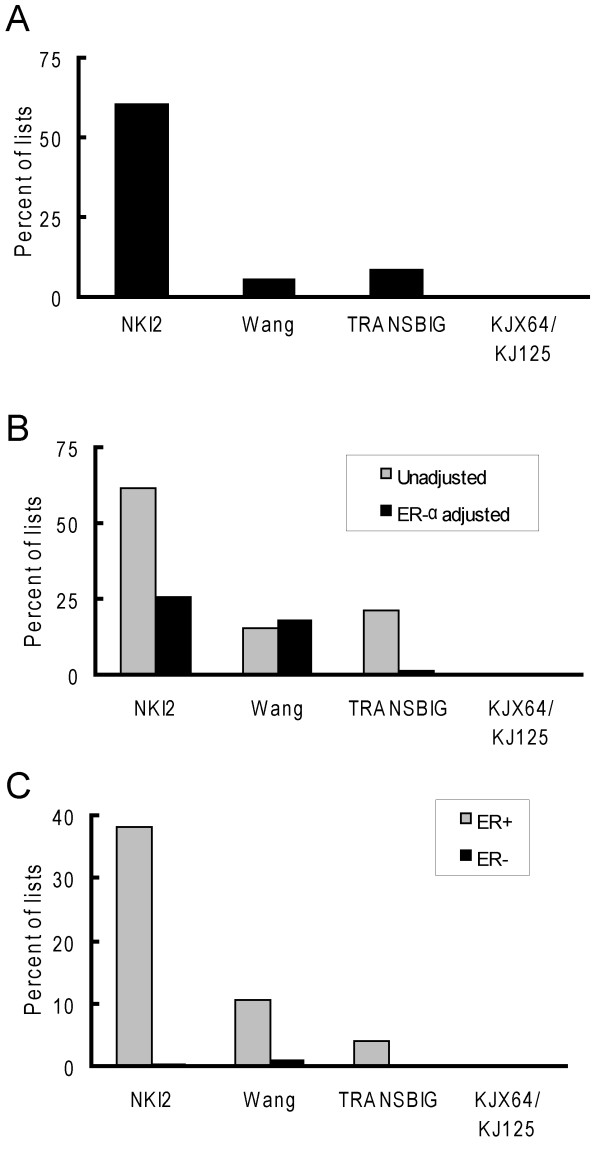
**Random genes lists have disparate prognostic frequencies across breast cancer gene expression data sets**. Each graph is a histogram showing the percentage of random gene lists (70 genes/list) that were significantly predictive of metastatic recurrence latencies in 4 gene expression data sets. A. The bars represent analyses based on 1,000 random lists of 70 genes derived from all genes within a data set. B. Cox regression analyses based on 5,000 random gene lists (70 genes/list) selected from 11,318 common unigenes IDs for either a univariate or multivariable model adjusting for expression of the ER-α gene. C. Analyses of tumors stratified by ER status based on 5,000 random gene lists (70 genes/list) selected from 11,318 genes that had a unique unigene identifier common to all 4 data sets. The same 5,000 lists were separately evaluated in either ER positive (ER+) or ER negative (ER-) tumors within each data set.

We sought to ascertain whether there may be intrinsic bias among publicly available breast cancer gene expression data sets that would influence the likelihood of observing a significant difference in metastatic tumor recurrence latencies based on gene expression patterns of primary tumors. To address this question, we employed an empirical approach whereby we determined the probability of identifying groups of tumors with statistically significant differences in recurrence latencies using hierarchical clustering that was driven by randomly generated lists of genes. Four previously published data sets were examined. Marked differences in the ability to segregate good and poor prognosis tumors were observed between the data sets using randomly generated gene lists of various sizes (figure 1A and Additional File 1). The highest likelihood of obtaining a positive result was observed in the NKI2 [4] data set where 60.1% of 1000 random lists of 70 genes each were able to stratify tumors into two groups with significant differences (p < 0.01) in recurrence latencies (figure 1A). The lowest likelihood of obtaining a positive result occurred with the KJX64/KJ125 [6] data set, where none of the 70-gene lists generated clusters with a significant difference in recurrence latencies.

We next assessed whether the differences among the data sets was the result of differences in the content of the genes represented on the microarrays used in each study. We performed clustering analysis using a set of 5,000 random lists (70 genes/list) that were comprised of genes that were common to all four data sets (figure 1B). For this analysis, each data set was modified so that it included only 1 instance of a given gene. Consistent with our initial observations, there was a significantly higher likelihood of obtaining a significant result when examining recurrence latencies in the NKI2 data set (61.6%), as compared to the other data sets. For the Wang [1] and TRANSBIG [20] data sets, use of a data set with only a single instance of each gene resulted in an increase in the proportion of predictive gene lists to 15% and 21%, respectively. This discrepancy is explained by the fact that decreasing the number of redundant probes in these data sets increased the relative proportion of genes predictive of outcome. Thus, these data sets likely contain multiple probes for many genes that are not associated with metastases.

In light of the known association between estrogen receptor (ER) status and disease outcome [22] and the fact that expression of numerous genes is associated with ER status in breast cancer expression data sets [11,16], these findings could be due to cluster assignment dictated by the ERα-correlated gene expression network. Among the four data sets we evaluated, ER status was associated with significant differences in latencies in the NKI2 and TRANSBIG data sets (table 1). In multivariable Cox regression models adjusting for ER-α gene expression, there was a significant attenuation in the number of random gene lists that were predictive in the NKI2 and TRANSBIG data sets (table 1B). In the NKI2 set, there was a 60% reduction in the number of predictive gene lists. However, approximately 25% percent of the random gene lists were still predictive. In contrast, none of the gene lists in the TRANSBIG gene set were predictive after ER-α adjustment, suggesting that none of the significant gene clusters were measuring significantly more information than ER-α gene expression. The Wang data set was relatively unaffected by ER-α adjustment.

**Table 1:** ER status is predictive of metastasis in the NKI2 and TRANSBIG data sets.

			Recurrence Event^1^		Recurrence^2^ (ER+ vs ER-)	
Data set	ER+ (n)	ER- (n)	ER+ (%)	ER- (%)	HR	p-value
NKI2	225	70	24	47	0.4	<0.0001
Wang	209	77	32	35	0.8	0.5
TRANSBIG	136	64	13	28	0.4	0.005
KJX64/KJ125	149	34	22	29	0.7	0.4

1. The percentages indicate the proportion of patients that had metastases within 5 years, stratified by the ER status of their primary tumors.

2. Hazard ratios (HR) and p-values are based on Cox proportional hazards regression analyses and represent the changes in the risk of experiencing a metastatic event for ER positive tumors, as compared to ER negative tumors.

Gruvberger et. al. has suggested that prognostic gene lists be tested independently on ER positive and negative tumors in order to control for the effects of ER-α gene expression on tumor assignment [16]. We re-examined our prognostic lists separately in ER positive and negative tumors. While none of the lists were predictive amongst ER negative tumors, the NKI2 data set continued to show a significantly higher likelihood of giving a positive finding, as compared to the other data sets (figure 1C). Specifically, 38% of the lists were predictive in the NKI2 set versus 11% and 4% in the Wang and NKI2 data sets, respectively. Again, no random lists were predictive in the KJX64/KJ125 data set.

In summary, these results indicate that there is a large disparity in the likelihood of observing significant differences in metastasis risk among breast cancer data sets using arbitrary gene expression levels as a classifier. In particular, it was 4–5 times more likely to obtain a significantly predictive gene list in the NKI2 data set than the other data sets, even in stratified analyses examining ER positive tumors.

### Cluster assignment is associated with ER status

**Figure 2:**
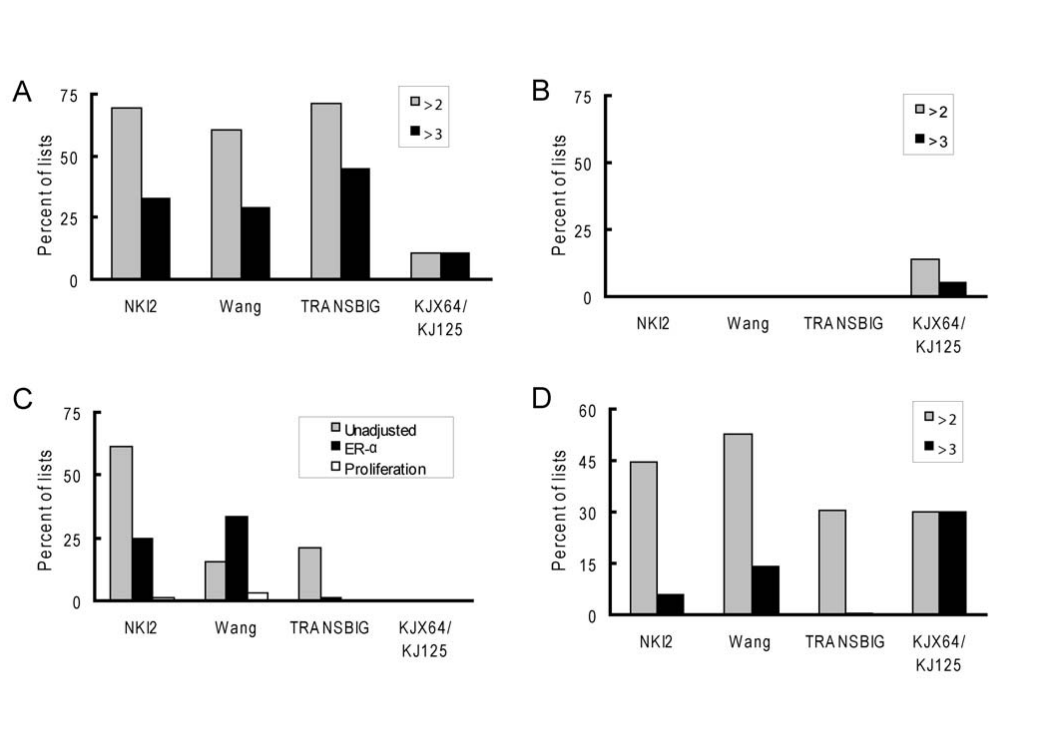
**Clustering associated with ER-α expression in the NKI2, Wang and TRANSBIG data sets**. All graphs were based on unsupervised hierarchical clustering analyses using the 5,000 randomly-generated unigene gene lists. A. Percentage of lists that segregate tumors into groups that contain a disproportionately high number of ER negative tumors as compared to ER positive tumors for each data set. A ratio greater than 1 indicates that the clusters contain a disproportionate number of ER negative tumors, as compared to ER positive tumors. The grey bars and black bars show the percentage of gene lists where this ratio is 2 and greater than 3, respectively. B. Percentage of lists that segregate tumors into groups that contain a disproportionately high number of ER negative tumors as compared to ER positive tumors in data sets that were globally adjusted for ER-α expression. C. Percentage of lists that were significantly predictive of metastatic recurrence latencies in unadjusted data sets (grey bars) or data sets that were globally adjusted for either ER-α expression (black bars) or a marker of proliferation (white bars). D. Percentage of lists that segregate tumors into groups that contain a disproportionately high number of ER negative tumors as compared to ER positive tumors in data sets that have been globally adjusted for a marker of proliferation.

The fact that multivariable adjustment for ER-α decreased the number of predictive gene lists in the NKI2 and TRANSBIG data sets suggests that the hierarchical clustering algorithm is functioning in large part by stratifying tumors into groups based on ER status. To confirm this, we examined the association between ER status and cluster assignment across the data sets. Specifically, we determined if there were disproportionate proportions of ER negative, as compared to ER positive, tumors in the different prognostic groups that were produced by the 5,000 random gene lists (70 genes/list) (figure 2A). In the NKI2, Wang and TRANSBIG data sets, tumors were frequently assigned to groups with disproportionate numbers of ER positive and negative tumors with approximately equal frequency, though the TRANSBIG data set had a higher proportion of clusters with a 3 or more fold difference in the proportion of ER negative tumors in one of the cluster pairs. A far lower proportion of the lists in the KJX64/KJ125 data set were skewed with respect to ER status. Based on these results, stratification by ER status and the established significant association between ER status and the risk of metastasis likely underlies the high proportion of predictive lists in the NKI2 and TRANSBIG data sets.

While clustering is associated with ER status, it is not clear whether this association is due to the specific contribution of ER-α correlated genes, as has been suggested [16], or whether other gene clusters associated with ER status may be driving this association. To directly ascertain the contribution of ER-α correlated genes to cluster assignment, the gene expression data were globally adjusted to eliminate the correlations between all genes in a data set and the ER-α gene [14,23]. To accomplish this, we fit a least-squares regression line to each probe in the dataset, using ER-α as the independent variable, and then computed the residual. This residual represented the new expression value for that probe. After adjustment, the tumors were reclustered using the previously generated 5,000 lists of 70-gene lists and reanalyzed. Global adjustment eliminated the propensity for clusters to be associated with ER status in all data sets except the KJX64/KJ125 data set, in which there was only a modest attenuation (figure 2B). This anomaly may be due to the fact that we observed that ER-α gene expression was poorly correlated with ER status in the KJX64/KJ125 data set (data not shown), as compared to the other data sets. ER-α adjustment of the NKI2 and TRANSBIG sets showed a similar attenuation in the proportion of predictive gene lists observed using multivariable adjustment for ER-α. This indicates that ER-α correlated genes make a significant contribution to the efficacy of the predictive gene lists in these data sets (figure 2C). In contrast, ER-α adjustment substantially increased the proportion of lists that were predictive in the Wang data set from 15% to 34%. This observation is likely explained by the fact that ER status is not associated with outcome in this data set. Hence, removing the constraint on the clustering algorithm to assign tumors to clusters associated with ER status increased the influence of other sets of correlated genes associated with prognosis on the clustering program. Adjustment did not have any impact on the KJX64/KJ125 data set.

In summary, these data show that ER-α correlated genes can serve to either increase or decrease the likelihood that a random set of genes will cluster tumors into groups with significant differences in latencies. The differences in the direction of the effect are related to the underlying association between ER status and the risk of metastasis in each data set.

### Proliferation-correlated genes eliminate the prognostic contributions of ER-α correlated genes

It has been previously shown that genes associated with cellular proliferation are strong predictors of metastases [13,14,24-26]. To ascertain the impact of adjusting for proliferation-correlated genes on cluster assignment and outcome, each data set was globally adjusted for proliferation. Adjustment almost completely eliminated the prognostic abilities of all of the random gene lists in the NKI2, Wang and TRANSBIG data sets (figure 2C), confirming the prognostic efficacy of the proliferation gene cluster. Interestingly, while adjustment for proliferation decreased the rate of disproportional cluster assignment associated with ER status, especially in the TRANSBIG data set, it did not eliminate it (figure 2D). Thus, the prognostic ability of the gene lists can be eliminated while maintaining a predilection for cluster assignment associated with ER status. These results suggest that the prognostic contribution of ER-α correlated genes can be explained by their correlation with proliferation genes.

### The relationship between ER-α and proliferation varies across data sets

**Figure 3:**
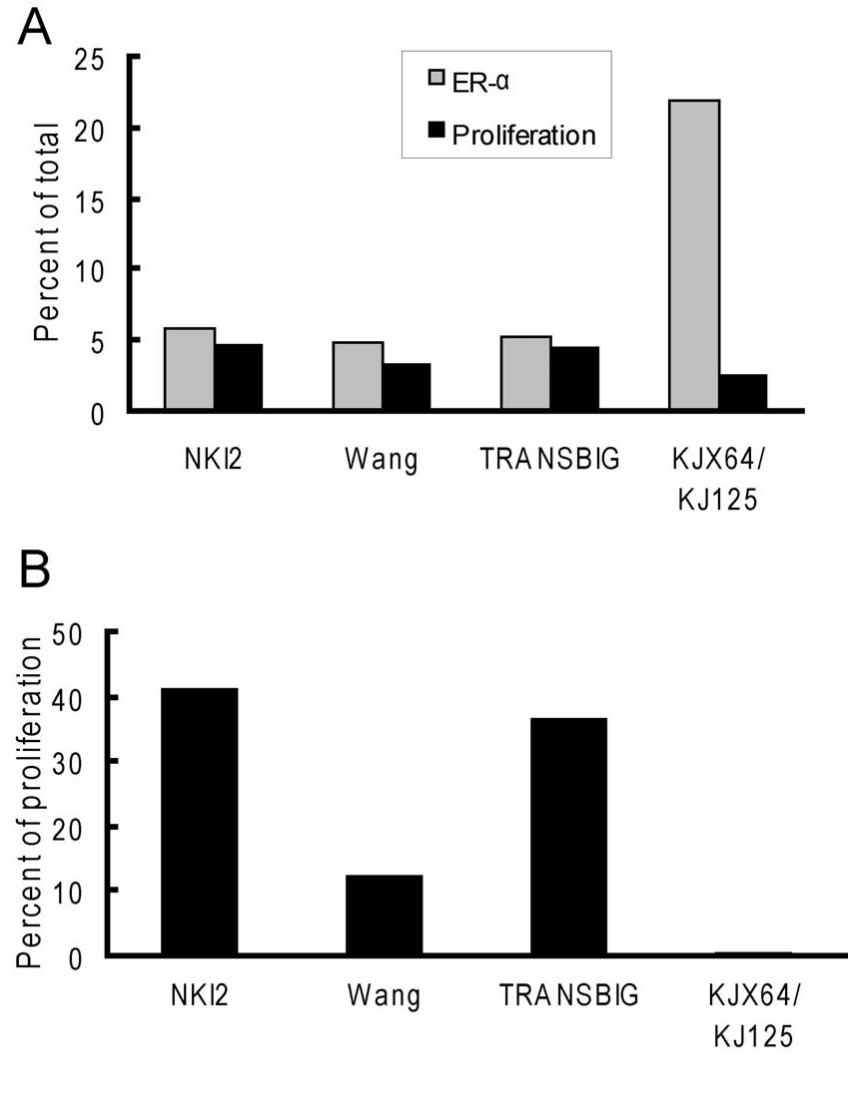
**ER-α expression account for more variance in the expression of proliferation markers in the NKI2 and TRANSBIG data sets**. A. The graph shows the percentage decrease in the sum of the variances of all genes within a data set after globally adjusting that data set for either ER-α expression (grey bars) or a marker of proliferation (black bars). B. The graph shows the percentage of the variance associated with adjustment for proliferation that is accounted for by adjustment for ER-α expression. This percentage reflects the extent to which genes whose expression follows proliferation-associated genes also fluctuate in response to changes in ER-α expression.

While global adjustment for proliferation eliminated the predictive capacity of the random gene lists in the NKI2, Wang and TRANSBIG data sets, adjustment for ER-α had differential effects across these data sets. To better characterize the relationship between genes that are correlated with ER-α or proliferation and cluster assignment, we characterized the inter-relationships between these gene clusters. We first examined the proportion of the total variance in each data set accounted for by adjustment for either ER-α or proliferation correlated genes (figure 3a). In the NKI2, Wang and TRANSBIG data sets, the genes correlated with each of these factors accounted for approximately similar amounts of the total variance in the data sets. This proportion was significantly higher for ER-α correlated genes in the KJX64/KJ125 data set. In all sets, ER-α correlated genes accounted for a larger proportion of variance than proliferation-correlated genes. For instance, in the NKI2 data set, ER-α adjustment accounted for 5.9% of the total variance versus 4.6% explained by proliferation genes. The higher proportion of variance accounted for by ER-α correlated genes, as compared to proliferation genes, allows these genes to be more influential in determining cluster assignment, as compared to proliferation genes. Surprisingly, while ER-α correlated genes explained a larger proportion of the variation in the KJX64/KJ125 data set, they did not promote clustering by ER status in this data set. Again, this is likely due to the poor correspondence between ER-α gene expression and ER status in this data set.

It is important to note that the variation explained by the ER-α and proliferation-correlated genes is not independent. Thus, in contrast to the above similarities between the data sets, the proportion of the proliferation-associated variance that is explained by ER-α differs substantially (figure 3B). In the NKI2 and TRANSBIG data sets, ER-α accounted for approximately 35–40% of the proliferation-associated variance. This relatively high proportion likely explains the significant association between ER status and prognosis and the impact of adjusting for ER-α on the prognostic abilities of the random gene lists in the NKI2 and TRANSBIG data sets. In the Wang data set, ER-α expression only accounted for 12% of the proliferative variance, hence explaining the weak association between ER status and prognosis.

### Proliferation-correlated genes do not explain disparities in class prediction among the data sets for ER positive tumors

**Figure 4:**
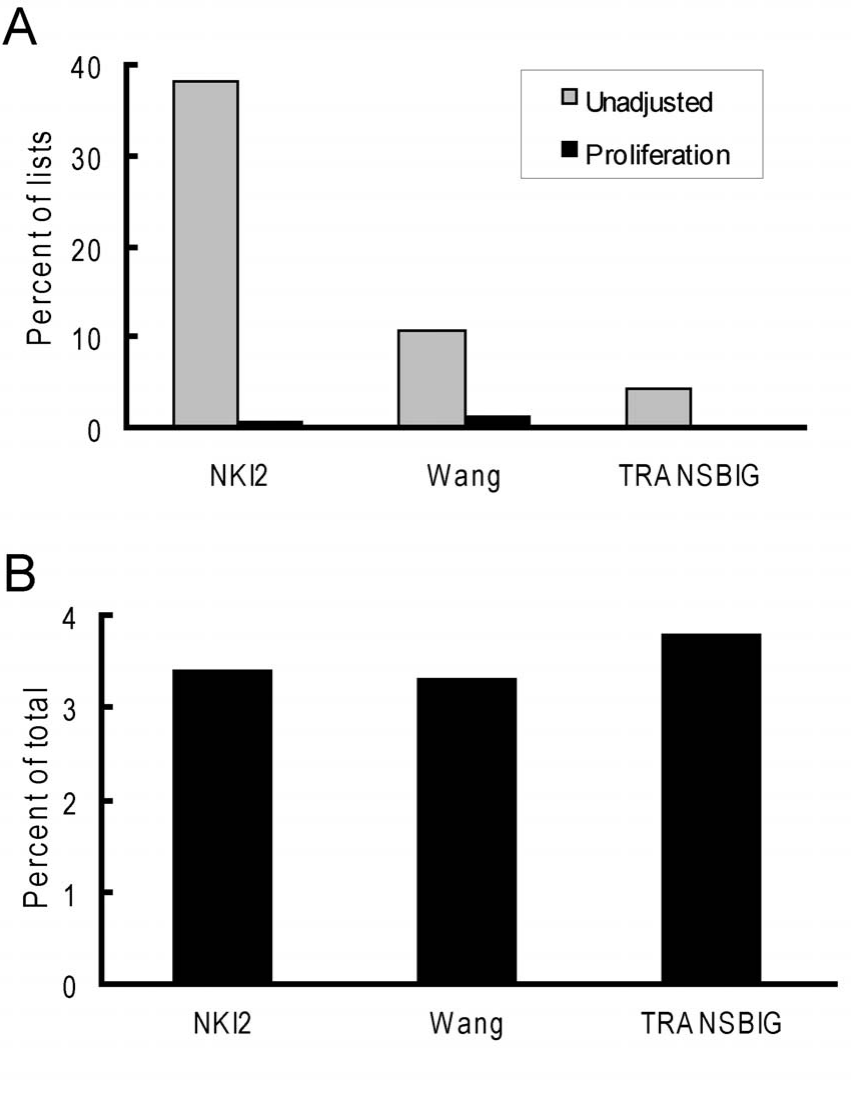
**ER positive tumors depend upon proliferation genes for cluster assignments with significant differences in latencies**. A. This graph was based on unsupervised hierarchical clustering analyses using the 5,000 randomly-generated unigene gene. The graph shows the percentage of lists that were significantly predictive of metastasis recurrence latencies in ER positive tumors in either unadjusted data sets (grey bars) or data sets that were globally adjusted for proliferation (black bars). B. The graph shows the percentage decrease in the sum of the variances of all genes in ER positive tumors after globally adjusting each data set for a marker of proliferation.

To gain more insight into possible differences between the NKI2, Wang and TRANSBIG data sets, we next examined the ER positive tumors. Amongst ER positive tumors, a random gene list was substantially more likely to give a significant result in the NKI2 data set, as compared to the other data sets. Among ER positive tumors, global adjustment for proliferation also eliminated the prognostic abilities of all of the random gene lists in the NKI2, Wang and TRANSBIG data sets (figure 4A). The consistency across data sets demonstrates that each of these data sets relies on a common feature to produce a significantly different prognostic cluster. Based on this observation, one explanation for the differences in frequency of positive findings across the data sets is that the proliferation-correlated genes account for a larger proportion of the total variance in ER positive tumors in the NKI2 data set. When the total variance explained by the proliferation genes was measured, it was found to be approximately equal in each of these data sets, though slightly higher in the TRANSBIG data set (figure 4B). Thus, the contribution of this predictive cluster of proliferation-associated genes to tumor assignment would be expected to be approximately equal in each data set and likely does not contribute to the highly increased probability of obtaining a positive result among ER positive tumors in the NKI2 data set.

### A larger sample size may account for differences between the NKI2 and TRANSBIG data sets

**Figure 5:**
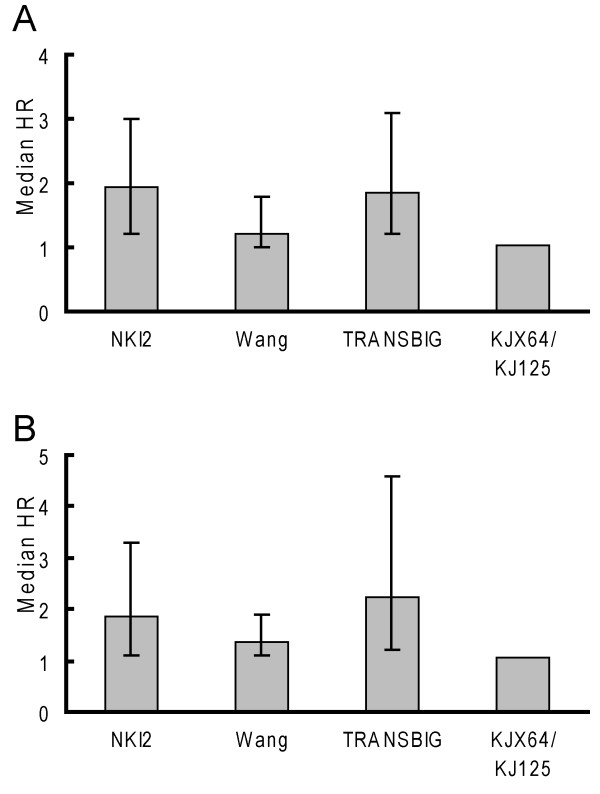
**Hazard ratio point estimates tend to be larger in the TRANSBIG data set for ER positive tumors**. The graphs were based on unsupervised hierarchical clustering analyses using the 5,000 randomly-generated unigene gene. Bars represent the median hazard ratio (HR) from univariate Cox regression analyses and the whiskers represent the inter-decile range (10%–90%) of the values. A. Results from analyses based on all tumors in each data set. B. Results from analyses based on ER positive tumors.

Another explanation for the disparities in the probability of identifying significantly different classes of tumors with random gene classifiers between the data sets, especially the NKI2 and TRANSBIG data set which share many commonalities, is differences in the sample sizes (table 1). The NKI2 and Wang data sets are considerably larger than the TRANSBIG data set. A larger sample size typically gives smaller standard errors of measurement and, consequently, smaller p-values. Thus, rather than using a p-value as an outcome, we examine hazard ratio (HR) coefficients, which show the magnitude of the differences in risk between cluster pairs, independent of sample size. When looking at all tumors in the NKI2, Wang and TRANSBIG data sets the median size and range of the HRs for the NKI2 and TRANSBIG data sets were approximately comparable (median of 2.0 vs. 1.9, respectively) (figure 5A). The median was only 1.2 in the Wang data set. However, when looking at only ER positive tumors, the TRANSBIG had the largest HR median value and range of the three data sets (figure 5B). Median values were 1.9, 1.4 and 2.2 in the NKI2, Wang and TRANSBIG data sets, respectively. There was very little variation in the range of HR values in the KJX64/KJ125, suggesting that, regardless of the random gene list utilized, the clustering algorithm created virtually identical clusters of tumors for each gene list. Overall, these data suggest that the larger number of significantly predictive random gene lists seen in the NKI2 versus TRANSBIG data set is likely the result of the larger sample size of the NKI2 data set.

## Discussion

In the present study, we show that there are marked differences in the likelihood of observing a positive association between the expression patterns of a set of genes and metastasis across four breast cancer global gene expression data sets. Most striking was the observation that over 60% of randomly selected lists of 70 genes each could segregate tumors into two groups with significant differences in outcome in the NKI2 data set. This finding is particularly salient in light of the fact that this data set has been frequently used to both generate and verify prognostic lists of genes [4,6,19,27-29]. The ability of the majority of random gene lists to predict outcome calls into question the biological validity of obtaining a positive finding in this data set, as a vast number of combinations of likely biologically unrelated genes are predictive of metastasis.

Gruvberger et. al., noting a strong association between ER status and outcome in the van't Veer data set [15], suggested that analyses of prognostic gene lists be stratified by ER status to ascertain whether the lists were simply functioning as proxies of ER-α gene expression [16]. ER status was associated with the risk of metastases in two of the data sets that we analyzed, the NKI2 (a partial superset of the van't Veer data set) and TRANSBIG data sets. In three of the four data sets, we found that tumors tended to cluster by ER status. This was directly attributable to genes correlated with ER-α expression, as elimination of the variance in gene expression associated with ER-α expression completely attenuated disproportionate clustering by ER status. Adjusting for the expression of ER-α decreased the proportion of predictive gene lists in the NKI2 and TRANSBIG data sets, as would be expected. It also substantially increased the proportion of predictive gene lists from approximately 15% to 35% in the Wang data set where ER status is not an independent predictor of metastasis. Thus, a gene list which gives disparate results in the Wang versus NKI2 or TRANSBIG data sets may, indeed, be functioning as a proxy for ER expression.

While ER-α expression may be an important contributor to cluster assignment, it is not the primary determinant for obtaining a significant prognostic gene list. When each of the data sets was globally adjusted for proliferation, there were almost no significantly predictive gene lists, even though tumors tended to cluster by ER status. Genes associated with proliferation have been shown to be the essential contributors to an effective prognostic gene list in gene expression data sets of combined ER positive and negative tumors or just ER positive tumors [13,14,26]. We show that the extent to which adjusting for ER expression impacts a prognostic gene list is related to the extent to which the expression the ER-α gene and proliferation genes covary. Hence, ER status is predictive of prognosis to the extent to which it functions as a confounder to the relationship between the expression of proliferation genes and the risk of metastasis.

Stratifying our analyses by ER status had differential effects on the data sets. In the NKI2 data set, almost 40% of gene lists were predictive in ER positive tumors, while less than 5% were predictive in the TRANSBIG data set. This result is interesting since both data sets had a similar proportion of events occurring among ER positive and negative tumors and ER status was associated with outcome in both data sets. When we examined the distribution of the hazard ratio estimates, which show the magnitude of the differences in rates of metastases of the tumors in the cluster pairs, the hazard ratio estimates tended to be larger in the TRANSBIG data set. This analysis suggests that clusters generated with the random gene lists tend to have larger differences in the metastasis latencies in the TRANSBIG data. Thus, the most likely explanation for the difference in the proportion of significant findings between these data sets is the larger sample size of the NKI2 data set. Other than sample size variations, these data sets were similar in many regards. The similarities may reflect commonalities in their patient populations, both of which were derived from young women in northern European countries.

In contrast to the ER positive tumors, virtually none of the random gene lists was prognostic in ER negative tumors in stratified analyses. This observation may suggest that, among these data sets, there is not a large, dominant cluster of correlated genes associated with prognosis among these tumors. Another possible explanation is that there is more heterogeneity among ER negative tumors, which can be comprised on Her2 amplified tumors, basal as well as familial forms of breast cancer [30-32]. We would note, however, that gene-expression based prognostic signatures for ER negative tumors have been described [33,34], suggesting that robust common prognostic features likely exist amongst these tumors.

We found that, in general, random gene lists were less predictive of recurrence in the Wang data set. In analyses of all tumors, this effect could be partially attributed to the lack of an association between ER status and outcome in this data set, as described above. The random gene lists were also far less predictive among ER positive tumors in this data set, as compared to the NKI2 data set. This difference cannot be attributed to sample size differences as these data sets are approximately the same size. Like the TRANSBIG data set, the Wang data set is comprised of lymph node negative patients. While this difference could account for disparities between the Wang and NKI2 data sets (which utilized tumors from patients with node positive and negative disease), it would not account for differences between it and the TRANSBIG data set. Thus, the relevance of the difference in tumor composition with respect to this clinical variable is not clear. Another notable difference between the data sets is the older age representation among participants in the Wang study which could contribute additional heterogeneity amongst these tumors. It is also interesting to note that random gene lists containing genes that were independently predictive of metastases were only predictive approximately 55% percent of the time in this data set, versus 90% or more of the time in the other data sets (see Additional File 1). This might suggest that there are more independent clusters of genes predictive of prognosis in this data set. Consistent with this hypothesis, we have previously found a higher degree of correlation among independently predictive genes in NKI2 data set versus the Wang data set [14]. Michiels *et. al*. showed in the van't Veer data set that the set of genes selected for a prognostic classifier is highly variable depending upon the training set used to identify prognostic genes [35]. Based on our findings, we would anticipate an even greater degree of variability in the genes identified if their analysis was repeated using the Wang data set.

The KJX64/KJ125 data set produced considerably different results than the other data sets. Virtually none of the random gene lists were prognostic in this data set. It is important to note that it is possible to generate a large set of prognostic genes lists in this data set, as we found that 97% of random gene list derived from genes independently associated with metastasis were indeed predictive (see Additional File 1). In addition, this data set has also been used to generate other validated gene lists [6]. We found anomalies in this data set that may account for differences in its behavior, as compared to the other data sets, such as the fact that the ER-α gene probe levels were poorly correlated with ER status. This might suggest that there are issues with scaling of the raw data or that there are a large proportion of gene probes which did not perform optimally. However, regardless of how we rescaled the data, we obtained nearly identical results. Furthermore, this data set was derived using the same gene expression technology (Affymetrix) as the TRANSBIG and Wang data sets. Hence, differences in behavior cannot be attributed to differences in technology.

## Conclusion

In summary, these analyses demonstrate that there are systematic differences among the four breast cancer gene expression data sets examined herein. Most notable is the fact that the NKI2 data set provides a very high likelihood of obtaining a significant association between expression and metastases for a given set of genes. This disparity persists when examining all tumors in the data set or when looking only at ER positive tumors. The TRANSBIG data set behaved similarly to the NKI2 data set and the lower likelihood of obtaining a positive result in this data set is likely attributable to its smaller sample size. Fewer random gene lists are predictive in the Wang data set due to the lack of an association between ER status and the risk of metastasis. These differences in data sets should be considered when using them for developing new prognostic classifiers or assessing the robustness of classifiers that were generated with other data sets.

## Competing interests

The authors declare that they have no competing interests.

## Authors' contributions

JDM and RAK contributed equally to the design and implementation of this study. All statistical analyses were performed by JDM. All authors read and approved the final manuscript.

## Pre-publication history

The pre-publication history for this paper can be accessed here:

http://www.biomedcentral.com/1471-2407/9/214/prepub
